# Differentiation potential of neural stem cells derived from fetal sheep

**DOI:** 10.1080/19768354.2017.1354915

**Published:** 2017-08-03

**Authors:** Qian Li, Shuang Zhang, Yanjie Zheng, Hebao Wen, Xiao Han, Minghai Zhang, Weijun Guan

**Affiliations:** a College of Wildlife Resources, Northeast Forestry University, Harbin, PR China; b Scientific Experiment Research Center, Harbin Institute of Physical Education, Harbin, PR China; c Institute of Animal Science, Chinese Academy of Agricultural Sciences, Beijing, PR China

**Keywords:** Cell culture, differentiation potential, fetal sheep, neural stem cells

## Abstract

Neural stem cells (NSCs) are multipotent stem cells that can differentiate into many cell types in vitro. In this study, we isolated and established an NSC line from fetal *Ovis aries*. Based on the results of immunofluorescence staining, NSCs expressed Nestin, Pax6 and MAP2. Moreover, a reverse transcription–polymerase chain reaction assay was used to biologically characterize the cell line. NSCs were induced to differentiate into neurogenic cells in vitro. They expressed MAP2, glial fibrillary acidic protein (GFAP) and myelin basic protein (MBP). In this study, we successfully isolated and cultivated NSCs from the hippocampal tissue of fetal sheep. NSCs not only displayed a self-renewal capacity but also had the potential to differentiate into neurons and glial cells. This study provided valuable experimental data for NSC transplant research.

## Introduction

Neural stem cells (NSCs) are mother cells with differentiation potential and a self-renewal capacity that can produce neural tissue by unequal division. They retain the multipotent capacity until they differentiate into a variety of cell types in response to microenvironmental cues (Ao et al. 2007; Mishina & Snider 2014). NSCs have been detected in the dentate gyrus of the hippocampus, the subventricular zone (SVZ) and olfactory system and have been isolated from non-neurogenic zones, such as the septum, striatum (Palmer et al. 1995), spinal cord, cerebral cortex, corpus callosum, optic nerve (Palmer et al. 1999) and eye (Tropepe et al. 2000). The characterization of NSCs isolated from the hippocampus of fetal sheep is the focus of the present study.

In this study, we obtained a group of cells that expressed the markers Nestin, Pax6 (paired box 6) and MAP2 (microtubule-associated protein 2). After prolonged cultivation, a few cells in the subpopulation spontaneously differentiated into neurons, astrocytes and oligodendrocytes (Morshead et al. 1994; Palmer et al. 1997; Takahashi et al. 1999; Song et al. 2002). Conventional drug treatment is unsatisfactory because drugs do not have the ability to activate the function of nerve cells. Therefore, the transplantation of NSCs in vitro is virtually the only effective method for treating neurological diseases. NSCs secrete a variety of neurotrophic factors to promote the restoration of damaged cells, strengthen the contacts among synapses and create new neural circuits. Thus, an NSC line is considered one of the best strategies in regenerative medicine, due to the ready accessibility of donor tissue (Krejci and Grim 2010).

## Materials and methods

### Experimental animal

A 2- to 3-month-old fetal sheep was provided by the Animal Experimental Base Institute of Animal Sciences, Chinese Academy of Agricultural Sciences, Beijing. Animal experiments were performed in accordance with the guidelines established by the Institutional Animal Care and Use Committee at Chinese Academy of Agriculture Sciences.

### Isolation and culture of NSCs

The hippocampus was isolated from the subgranular zone (SGZ), and then vessels and debris were removed in 1 M phosphate-buffered saline (PBS). The tissue was rinsed 6–10 times with PBS and the cells were dispersed by repeated pipetting. Then, the cells were filtered with a 200-mesh sieve. Cells were centrifuged at 200 g for 7 min and then resuspended in Dulbecco’s Modified Eagle’s Medium/Ham’s F-12 (D/F12) medium (Gibco, Carlsbad, CA, USA) to which 20 ng/ml epidermal growth factor (EGF; Peprotech, Rocky Hill, TX, USA), 20 ng/ml basic fibroblast growth factor (bFGF; Peprotech), B27 supplement (1:50, Gibco), N2 supplement (1:50, Gibco), 1 M heparin, 2 mM glutamine, 100 U/ml penicillin and 100 mg/ml streptomycin had been added. Cells were incubated in a 37°C incubator with 5% (v/v) CO_2_. The medium was changed when the cells grew as floating neurospheres after 7–8 days (Krejci & Grim 2010). Subculturing was performed by repeatedly pipetting the cells followed by centrifugation at 750 g for 10 min. Half of the medium was changed every 4 days. The number of cells was recorded at 2, 4 and 7 days after the initial seeding. The morphology and growth of NSCs were observed under an inverted microscope.

## Differentiation of NSCs

Fifth-generation NSCs were cultivated in laminin/poly-L-lysine-coated 6-well plates or 12-well plates for 12 h. Then, cells were covered with D/F12 containing 5% fetal bovine serum (FBS; Gibco), 10 ng/ml EGF, 20 ng/ml bFGF, penicillin/streptomycin and L-glutamine for 3 h in an incubator to allow the spheres/clusters to attach to the coverslips (Li et al. 2007). Differentiation was induced by a combination of neurotrophins, including 10 ng/ml brain-derived neurotrophic factor (GDNF; Peprotech) and 10 ng/ml neurotrophin-3 (NT-3; Peprotech); the medium was changed every 4 days (Alexanian 2010). Differentiation was observed every day. The differentiation frequency depends on the culture conditions.

## Population doubling time and cloning efficiency

We selected P5 cells in good condition and plated them in 24-well plates at a density of 1 × 10^4^ cells ml^−1^. The population doubling time (PDT) was calculated in three randomly selected wells daily. Cell viability was detected using Trypan blue staining. The experiment lasted for 8 days. Culture time was plotted on the abscissa along with the ordinate cell number on the growth curve to calculate the PDT using the formula: PDT = (*t* − *t*0) lg2/ (lgNt − lgN0), where *t*0 is the initial culture time, *t* is the terminal culture time, N0 is the initial number of cultured cells and Nt is the terminal number of cultured cells.

We performed a clone analysis to detect the multipotency of NSCs. Single cells from the fourth or fifth generation were stained with Trypan blue, counted under a microscope, and seeded in 24-well plates at a density of 3000–4000 single cells per well. The growth of clone-spheres was observed and the number of colonies was counted. The cloning efficiency was calculated after cells were cultured for 6–7 days using the formula: Cloning efficiency (%) = (number of colonies/number of seeded cells) × 100%.

## Immunocytochemistry

Cells were fixed with 4% paraformaldehyde in 1% PBS for 20 min at room temperature and then washed three times. Cells were permeabilized with 0.25% Triton X-100 for 15 min. Nonspecific binding was blocked with 10% bovine serum albumin. Primary antibodies used for immunocytochemistry included: anti-Nestin (1:100, Santa Cruz Biotechnology, Inc.), anti-Pax6 (1:100, Santa Cruz Biotechnology, Inc.), polyclonal anti-MAP2 (1:100, Santa Cruz Biotechnology, Inc.), anti-GFAP (1:100, Santa Cruz Biotechnology, Inc.) and anti-MBP (1:100, Abcam, Cambridge, UK) antibodies. Cells were incubated with the antibodies overnight at 4°C. Secondary antibodies (conjugated with IgG or Cy5) were applied at a 1:100 dilution for 1 hour at room temperature. Anti-Pax6 and anti-MAP2 immunoreactions were visualized using a Cy5-conjugated secondary antibody (1:100; Zsbio Demo Store, Beijing, China). Anti-GFAP, anti-MBP and anti-Nestin immunoreactions were visualized using IgG.

## RNA analysis

Total RNA was isolated from secondary cells and extracted with Trizol using a one-step method for the reverse transcription–polymerase chain reaction (RT-PCR) analysis. The first strand cDNA was synthesized with PrimerSript Reverse Transcriptase (TaKaRa) and primers were designed and synthetized by Sangon Biotech (Sangon, Shanghai, China). The primers were designed to span the intron region to avoid false amplification. PCR cycling conditions were as follows: 5 min at 94°C followed by 30 cycles of 94°C for 30 s; 60°C or 62°C for 30 s; 72°C for 40 s and a final 5-min elongating step at 72°C. The length of the PCR fragments ranged from 200 to 300 bp. Primers used for RT-PCR are listed in Table 1.

**Table 1.** RT-PCR primer sequences of NSCs.

Gene	Direction	Primer sequences (5′→3′)	*T*m (°C)	Product length (bp)	Cycle number
Nestin	Forward	ACCCTTCCTGACTCCACTCC	60	232	35
	Reverse	CCTCAAACTCTTCCGACAGC			
Pax6	Forward	GACAACAACAAAGCGGACTG	60	222	35
	Reverse	TCACCCAACAAAGGCTCATT			
Notch1	Forward	GCGACAGCCTCAATGGGTA	62	204	35
	Reverse	GGTTGGACGCACACTCGTT			
VIM	Forward	CAGATGCGTGAAATGGAAGA	60	222	35
	Reverse	TGGAAGAGGCAGAGAAATCC			
CXCR4	Forward	GCCTGGTATCGTCATCCTGT	60	201	35
	Reverse	TCAAACTCACACCCTTGCTG			
GFAP	Forward	GTGGTGAAGACCGTGGAGAT	60	209	35
	Reverse	AGCAGGAAGAGGAGCAACTG			
S100β	Forward	CTTCTTTGGCTCGGACAGG	60	262	35
	Reverse	TTGCGATGGAGGAGGTGT			
MAP2	Forward	ACGCCAGGACTGCATCTTAT	60	231	35
	Reverse	TGGCTCAGGCTCTCTAGCTC			
TUBB3	Forward	CATCCAGAGCAAGAACAGCA	60	235	35
	Reverse	CTCGGTGAACTCCATCTCGT			
MBP	Forward	AGCTCACCCTTGGAGATCAG	60	256	35
	Reverse	CTGCCACGTACCAAAGCTC			
MOG	Forward	TCTCCAGGGTGGTTCATCTC	60	202	35
	Reverse	ATTGCTGCCTCCTCTTGGTA			
GAPDH	Forward	GCCGTAACTTCTGTGCTGTG	60	229	35
	Reverse	CGTTCTCTGCCTTGACTGTG			

## Results

### The ability of NSCs to agglomerate into clusters/spheres

**Figure 1.**
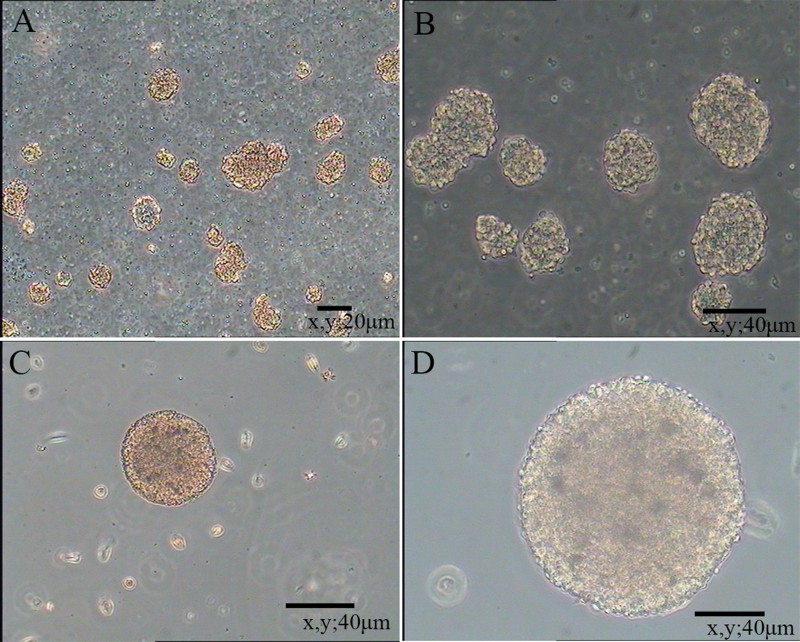
Floating neural spheres generated from hippocampal in serum-free medium. (A) and (C) were primary culture. (B) and (D) were secondary culture. (A) 10 μm, (B) 20 μm, (C) 40 μm and (D) 80 μm.

**Figure 2.**
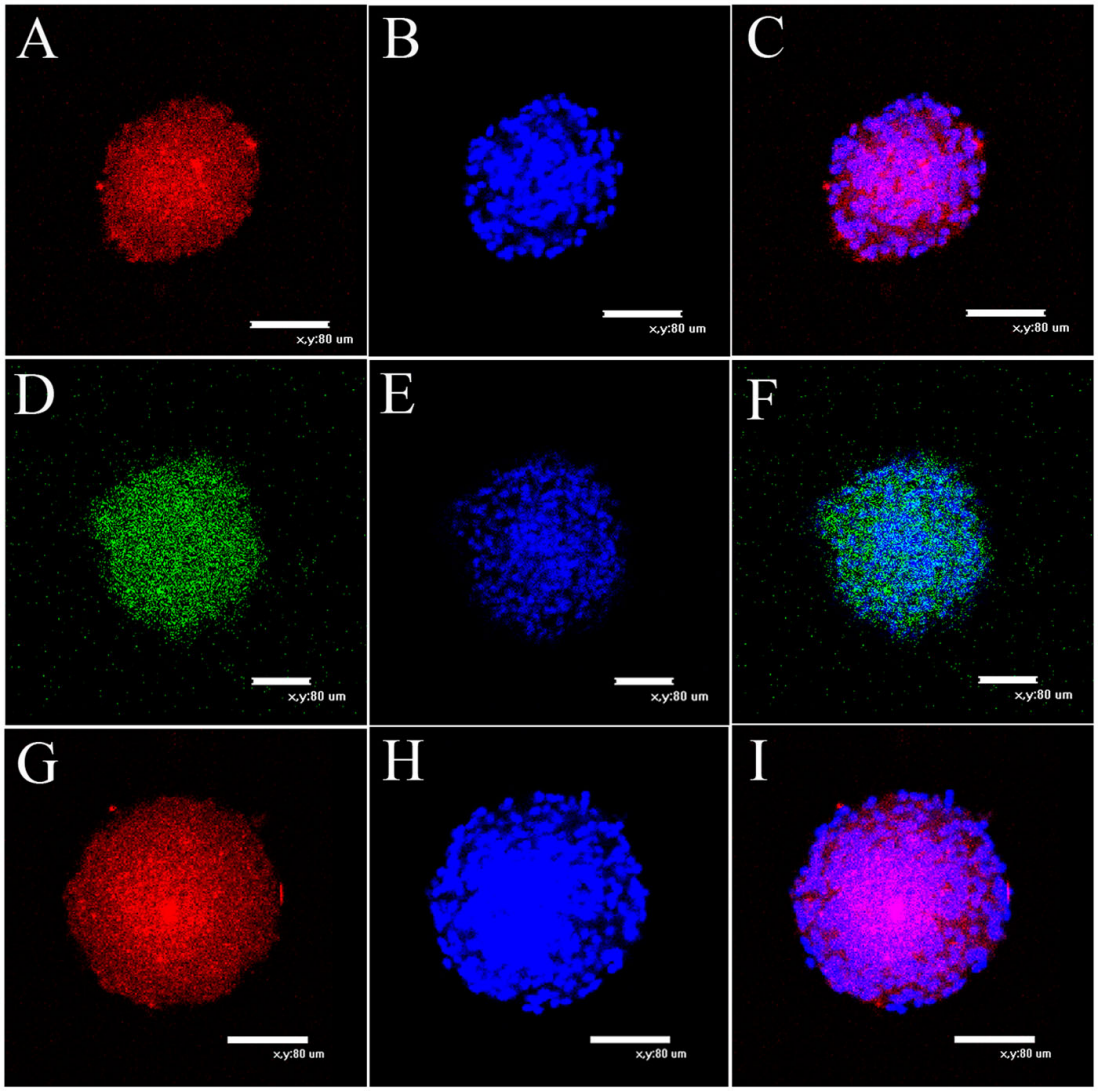
Immunocytochemical characterization of NCSCs. (A) Expression of NSCs marker MAP2. (C) Colocalization of nuclear stains DAPI and MAP2. (D) Expression of marker from immature and undifferentiated cells Nestin. (F) Colocalization of nuclear stain DAPI and Nestin. (G) Most of emigrated cells express stem cell marker Pax6. (I) Colocalization of nuclear stain DAPI and Pax6. (B), (E) and (H) are nuclear stain DAPI. Scale bars = 80 μm.

Primary NSCs were spherical and grew in suspension with a robust morphology, clear boundary and strong refraction. In the presence of mitogens such as EGF and bFGF, single cells agglomerated into dense clusters. Spheres were collected and dissociated into single cells to examine their self-renewal capacity. As shown in Figure 1, large numbers of single cells formed new spheres. Based on the results of immunofluorescence staining (Figure 2), NSCs expressed MAP2, Nestin and Pax6. Thus, NSCs exhibited the self-renewal capacity and were multipotent.

### RT-PCR detection

**Figure 3.**
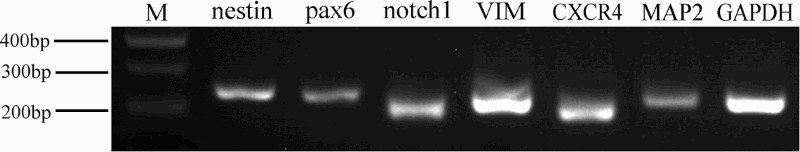
RT-PCR detection of partial NSCs and NSCs progeny markers. Lanes 1–7 show NCSCs expressing markers (Nestin, Pax6, Notch1, VIM, CXCR4, MAP-2) and expression of housekeeping gene GAPDH. M = 100 bp DNA ladder.

NSC and NSC progeny markers were used to retest NSC proliferation. As shown in Figure 3, NSCs expressed the Nestin, Pax6, Notch1, VIM, CXCR4 and MAP2 mRNAs.

### PDT and proliferation of NSCs

**Figure 4.**
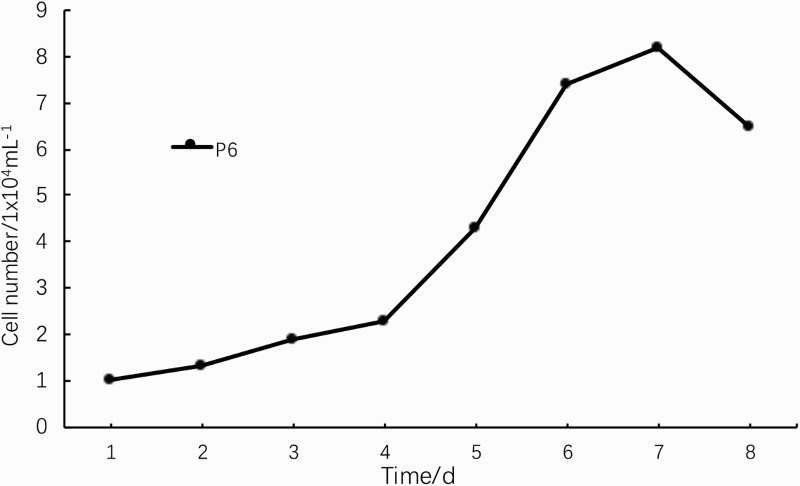
Growth curve of NSCs.

The growth curve of P5 cells shown in Figure 4 was an ‘S’ type curve. After 1 day of incubation, cells entered the logarithmic growth phase and proliferated rapidly. After 6–7 days, the cell growth rate was significantly reduced and cells entered the platform phase. Then, cell growth was inhibited and cells began to age (Figure 4). As the number of generations increased, cells grew slowly, were refractory and the sphere-forming ability decreased. The PDT was significantly prolonged and cell transparency and viability decreased beginning at passage 8.

**Figure 5.**
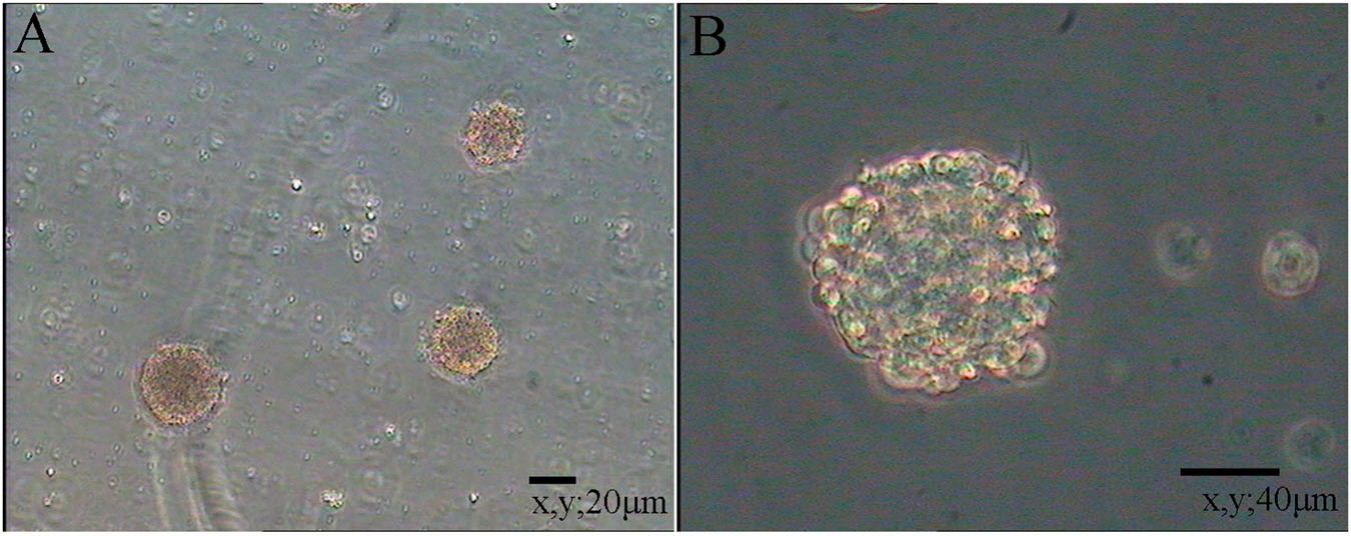
Cloning efficiency of NSCs.

Cloning efficiency was used to analyze the growth rates of NSCs. The small clusters were counted and observed after 6–7 days of culture (Figure 5). The average number of clusters in the 24-well plate was 200 ± 20 and the cloning efficiency was 7 ± 0.5%.

### NSCs differentiation and identification of neurogenic cells

**Figure 6.**
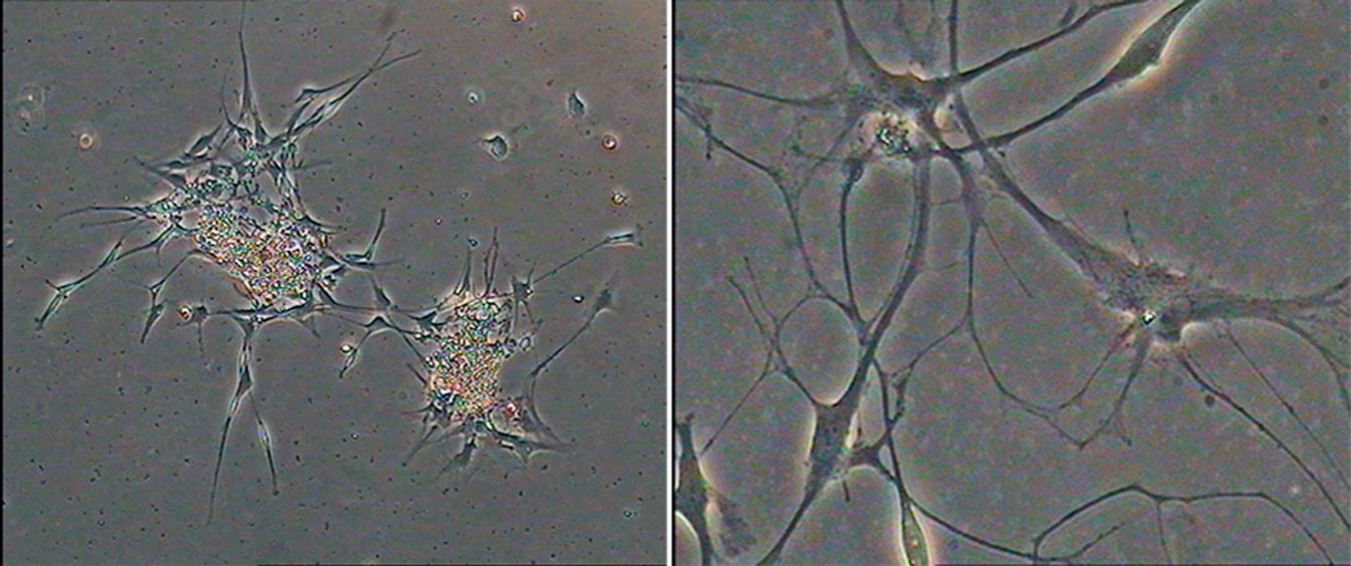
Differentiation of NSCs derived from hippocampus.

Cells began to adhere and extend short projections after 12 h (Li et al. 2010). Most of the cells continuously extended irregular protrusions. After a week, differentiated cells divided into multi-projection stellate cells and neuron-like cells, whose projections intertwined into a network (Figure 6). After 10 days, cells grew as a monolayer and differentiated into different types of cells.

The majority of NSCs differentiated into astrocytes and neural cells, whereas only a small proportion differentiated into oligodendrocytes. The differentiated cells were distinguished under a microscope. Neurons were small and round, with a regular morphology, a smooth edge and spindle, and 2–3 long projections. Neurons were clustered and the projections were interwoven into a network, which was located in the vicinity of the neurospheres. The proportion of MAP2^+^ cells among the total differentiated cells was approximately 51.6%. Astrocytes were large cells that extended many short projections in a star-shaped pattern. The cytoplasm was rich and the nucleus was large. The astrocytes were located at the bottom of the differentiated cells. The proportion of GFAP^+^ cells among the total number of differentiated cells was approximately 39.2%. Oligodendrocytes extended multiple projections and branches that were smaller than astrocytes, and the nucleus was round and small. Oligodendrocytes contained many microtubules. The proportion of MBP^+^ cells among the total number of differentiated cells was approximately 9.2%. Previous studies have reported different proportions of NSCs that differentiated into neurons and glial cells, possibly because of different culture and differentiation conditions.

**Figure 7.**
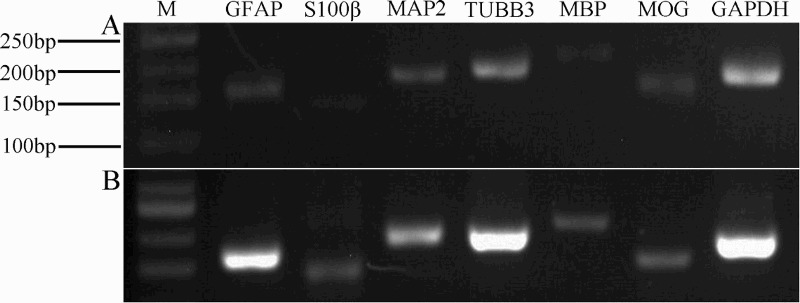
Molecular profile of the spheres differentiated products (B) Comparison of the NSCs (A). RT-PCR on selected genes was performed by using total RNA that isolated from NSCs and the induced cells derived from neural spheres. The representative bands of the PCR products ran in 2.5% agarose gel electrophoresis. RT-PCR for GAPDH was used as loading control. M = 50 bp DNA ladder.

**Figure 8.**
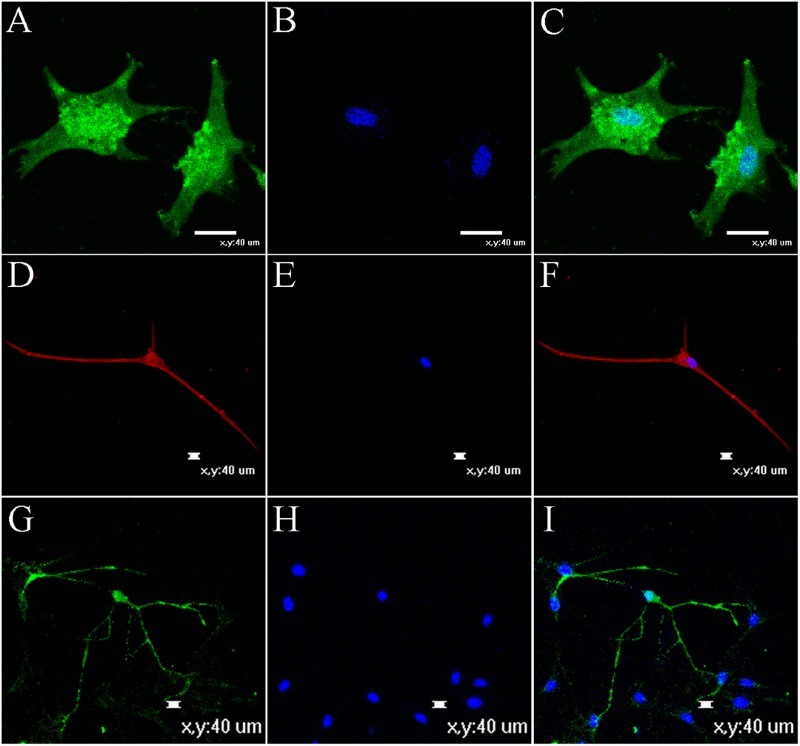
Neurotrophic factors promoted neuritogenesis and maturation of neurons and glia derived from NSCs after induced for 12 days in vitro. (A) and (B) GFAP expression and nuclear staining. GFAP as astrocyte maker. (C) Merger of (A) and (B). (D) MAP2, as neuronal marker. (F) Merger of (D) and (E). (G) MBP expression. MBP as oligodendrocyte maker. (I) Merger of (G) and (H). (E) and (H) Nuclear staining. Bar scales =  40 μm.

Some neurogenic cells began to die after 2 weeks. To confirm our immunocytochemical findings, we used RT-PCR to analyze the neurogenic cells and further investigate their properties. Specifically, we examined the expression of the GFAP, s-100β, MAP-2, TUBB3, MBP and MOG genes, which were all positive (Figure 7(A)); simultaneously, we compared the expression patterns in neurogenic cells with the expression patterns observed in NSCs (Figure 7(B)). Some genes that were expressed in the NSCs were also expressed in the differentiated cells. For example, MAP2 was expressed in both neurons and NSCs. Based on the results of the RT-PCR analysis, the two cell types displayed different expression levels for the same set of genes and the regional specificity of stem cells in the nervous system. However, the mechanism by which these genes control the differentiation of neural stem cells requires further study. Immunofluorescence staining for GFAP, MAP-2 and MBP showed positive expression (Figure 8).

## Discussion

NSCs are widely expressed throughout the mammalian central nervous system, including the lateral ventricle, striatum, hippocampus, olfactory bulb and spinal cord, but NSCs have also been detected in other tissues. The adult mammalian central nervous system has two highly dense zones of NSCs, namely the SVZ of the lateral ventricle and the SGZ of the hippocampal dentate gyrus (Doetsch et al. 1999; Laywell et al. 2000; Garcia et al. 2004). We aimed to identify NSCs based on three features, the ability to divide, pluripotency and the expression of specific markers, to determine whether the isolated cells cultured in vitro were actually NSCs. In this study, we obtained colonies from fetal sheep in vitro. Specific markers of NSCs were detected using immunofluorescence staining.

As the NSC research methods and techniques have constantly been updated, the characteristics and application of NSCs have recently made great progress. However, the basis and premise of the study and application are to successfully isolate and culture NSCs. Currently, in vitro cell culture techniques are more proficient. In this experiment, we used the mechanical separation method to obtain single cell suspensions from nervous tissue. The mechanical separation method is the most direct method of reducing the fiber content in brain tissue, embryonic tissue, etc. This method is simple, rapid and induces relatively little cell damage. The single cell suspension contains some cellular debris, but it had little effect on NSCs. Due to the use of a specific medium, other cells cannot grow and gradually die (Myckatyn et al. 2004).

Primary NSCs are mixed with nerve cells and glial cells. However, we need to isolate a single cell type to study its morphology and characteristics. Therefore, the purification of NSCs in vitro is a very important step. Currently, the main purification methods are the flow cytometry method, differential centrifugation and the selective medium method. The flow cytometry method generates NSCs with greater than 90% purity, but this method requires special equipment, a lengthy operating procedure and a greater number of uncontrollable factors (Nakamura et al. 2003). Differential centrifugation mainly uses differences in cell density to separate NSCs with different centrifugal speeds. However, centrifugation conditions are difficult to control and the centrifugal effect is uncertain (Okano et al. 2003). The selective medium method is one of the most commonly used methods for the purification of NSCs. It uses the nature of NSC proliferation in special culture medium to exclude other cells. In this study, we adopted the selective medium method. When primary cells were cultured in the medium for a defined period, other types of cells could not adapt to the culture environment and gradually died. The surviving cells continued to proliferate and formed neurospheres after a few days (Ao et al. 2007).

We added components such as EGF, bFGF and heparin to serum-free medium to promote growth and differentiation and to satisfy the different aims of our study. The different components have different functions in determining cell positions and developmental stages. Therefore, studies of the effects of various factors have become a hot topic in biological research (Wang et al. 2010).

Neurotrophic factors and the protective factor bFGF are important factors related to proliferation and differentiation. The growth factor bFGF plays a specific role during NSCs differentiation into glial cells and neurons (Janebodin et al. 2011). The main function of EGF is to promote the long-term survival of NSCs (Sutterlin et al. 2013). Different effects have been observed when EGF and bFGF are used alone or in combination, due to the different culture conditions (Shihabuddin et al. 1997). In most cases, proliferation requires at least two interacting factors. The mechanism of proliferation and the nature of spatial and temporal regulation are the focus of neurobiology studies.

In the differentiation experiments, we applied 5% FBS that contained various equilibrium factors (Shalaw et al. 2006). NSCs tend to differentiate. With the addition of GDNF, NT-3 and other nutritional factors, NSCs differentiate into two important cell types, astrocytes and neurons. Most NSCs differentiated into astrocytes and glial cells, and few differentiated into oligodendrocytes. Our study represents a preliminary study of NSC differentiation. Currently, the mechanisms by which NSCs proliferate and differentiate are unclear, as they are very complicated processes. Because NSC applications aim to use differentiated cells with different properties and functions (Ferroni et al. 2013), experiments designed to determine the functions of different factors in NSCs and the expression of genes and proteins will be the focus of future studies.

## Conclusions

In this study, we successfully isolated and cultured NSCs from the hippocampus of fetal sheep, identified cell morphology and assessed molecular biology. The self-renewal capability and differentiation potential of NSCs were assessed in vitro. NSCs were induced to differentiate into neurons, astrocytes and oligodendrocytes, indicating that NSCs are multipotent stem cells. Through this study, we not only provided a fundamental technology platform for the establishment of a fetal sheep NSC bank but also reported the potential application of NSCs as a stem cell source for regenerative therapies. Studies on NSC differentiation will improve the prospects for regeneration in patients with neurodegenerative diseases, such as Parkinson’s disease and Alzheimer’s disease, as well as screens of nervous system drugs. An optimal method to induce the complete differentiation of NSCs into a particular type of neuron is not currently available; therefore, further studies of neural stem cells are needed.
